# The cost-effectiveness of mental health interventions amongst prison populations: a systematic review (research letter to the editor)

**DOI:** 10.1080/14789949.2024.2350515

**Published:** 2024-05-10

**Authors:** Aleix Rowlandson, Gemma Shields, Elise Blakemore, Iniyah Sulaman, Charlotte Lennox, Rebecca Crook, David Honeywell, Daniel Pratt

**Affiliations:** a Manchester Centre for Health Economics, Division of Population Health, Health Services Research and Primary Care, School of Health Sciences, Faculty of Biology, Medicine and Health, University of Manchester, UK; b EPIC Learning and Psychology Service, Doncaster City Council, UK; c REVIVE Partnership, East Lancashire Teaching Hospitals Trust, Burnley, UK; d Division of Psychology and Mental Health, Faculty of Biology, Medicine and Health, Health and Justice Research Network, University of Manchester, UK; e Centre for New Treatments and Understanding in Mental Health (CeNTrUM), Division of Psychology and Mental Health, Faculty of Biology, Medicine and Health, University of Manchester, UK; f Public Health, Policy & Systems, Institute of Population Health, Faculty of Health and Life Sciences, University of Liverpool, UK; g School of Criminal Justice, Arden University, Coventry, UK; h Greater Manchester Mental Health NHS Foundation Trust, School of Health Sciences, Faculty of Biology, Medicine and Health, University of Manchester, UK

**Keywords:** Cost-effectiveness, prison populations, mental health, incarceration, systematic review

## Abstract

The link between imprisonment and adverse mental health is well established and linked to both recidivism and prison misconduct, with negative consequences for prisoners, the prison system and society. To help minimise these impacts, appropriate mental health interventions are required. However, owing to finite resources to deliver healthcare in prisons, interventions must be both clinically and cost-effective. A systematic literature search was conducted using various medical and economic databases. The search aimed to identify full economic evaluations (comparing costs and consequences of two or more interventions) of mental health interventions for adult prisoners during incarceration. Results were intended to identify evidence gaps and highlight areas for future research. Only one publication met all eligibility requirements, with several limitations identified. This finding highlighted a clear lack of cost-effectiveness evidence for use by decision makers within the prison setting. This emphasises the need for future research to incorporate economic evaluation during the early stages of research design. Research should aim to incorporate both intervention costs and wider healthcare resource use, which may be affected, and generic outcomes, such as quality-adjusted life years (QALYs), which enable comparison across various disease areas and against pre-determined thresholds.

Dear editor,

The link between imprisonment and mental illness is well documented (Baranyi et al., 2019; Bebbington et al., 2017; Gómez-Figueroa & Camino-Proaño, 2022; Rebbapragada et al., 2021; Tyler et al., 2019). An increased prevalence is reported among prisoners compared to general populations for all investigated mental conditions, with neurotic disorders (e.g. anxiety, depression and personality disorder) and substance abuse most prominent (Baranyi et al., 2019; Bebbington et al., 2017; Gómez-Figueroa & Camino-Proaño, 2022; Rebbapragada et al., 2021; Tyler et al., 2019). While conditions and predispositions may exist prior to incarceration, the prison environment (e.g. overcrowding, isolation, lack of personal control) can both trigger and perpetuate symptoms of mental illness, with longstanding effects found to persist post-release (Cunha et al., 2023; Gómez-Figueroa & Camino-Proaño, 2022).

Poor mental health among prisoners is linked to increased prison misconduct (Houser et al., 2012; Semenza & Grosholz, 2019) and recidivism, with negative impacts for inmates, prison institutions and society (Chang et al., 2015; Edgemon & Clay-Warner, 2019; Ogilvie et al., 2023). Recidivism places a significant financial burden on both society and the prison system, with the social and economic cost of reoffending estimated at £18.1bn in the UK for 2019 (Newton et al., 2019). Recidivism also contributes to overcrowding, a well-known risk factor for adverse mental health among prisoners, perhaps perpetuating the issue at hand (Edgemon & Clay-Warner, 2019). Additionally, where prisoners with adverse mental health are placed in secure mental health units, this generates a significant cost burden; with a cost per bed day of £739, versus an average cost per day of £86 among public sector and contracted prisons in 2021–2022 (Ministry of Justice, 2023; NHS England, 2023).

There is a critical need for effective mental health interventions within the prison service to minimise negative outcomes among prisoners who are most vulnerable due to adverse mental health. However, prisons often have limited resources available to deliver healthcare, with mental healthcare in particular noted as being underfunded and underdeveloped (Forrester et al., 2018; Jack et al., 2018). There is not only a moral obligation to ensure support for vulnerable prisoners, but as prisons are funded by society, there is also a need to ensure that public money is used both efficiently and effectively. To ensure the optimal allocation of the finite resources available to deliver healthcare in prisons, economic evidence is required to support decision-making. Economic evaluations which compare the costs and consequences of alternative programmes or interventions provide vital evidence on cost-effectiveness to inform decision-making (The National Institute for Health and Care Excellence [NICE], 2014).

Suicide rates among UK prisoners were previously reported at 3.9 times those of the general population (Office for National Statistics [ONS], 2023), highlighting a need for cost-effective interventions to address prisoners adverse mental health. A recent review by Knapp and Wong (2023) aimed to provide an update on economic evidence for mental health interventions across the criminal justice system and summarised that there is a paucity of high-quality economic evidence (Knapp & Wong, 2023). However, the review was narrative in focus and did not include a formal quality assessment.

The PROSPECT programme aims to improve treatment for prisoner patients at risk of suicide, promote patient access to a Cognitive Behavioural Suicide Prevention (CBSP) programme within prisons, and reduce the economic and social costs of inefficient or ineffective treatments (ISRCTN – ISRCTN14056534). As part of this research, a systematic review was conducted, with full details, including the search strategy and eligibility criteria, available online via the PROSPERO International prospective register of systematic reviews database (CRD42023414765) (Rowlandson et al., 2023). This review aimed to identify full economic evaluations (comparing costs and consequences of two or more interventions) of mental health interventions for adult prisoners during the incarceration period. This was important as whilst partial economic evaluations (e.g. costs alone or outcomes alone) may be available, they do not allow us to consider value for money. The review also aimed to formally appraise the quality of identified evidence and highlight research gaps (to inform future research), addressing one of the key limitations identified with the recent review by Knapp and Wong (2023). Figure 1 depicts the flow of studies through the review process.

**Figure 1.**
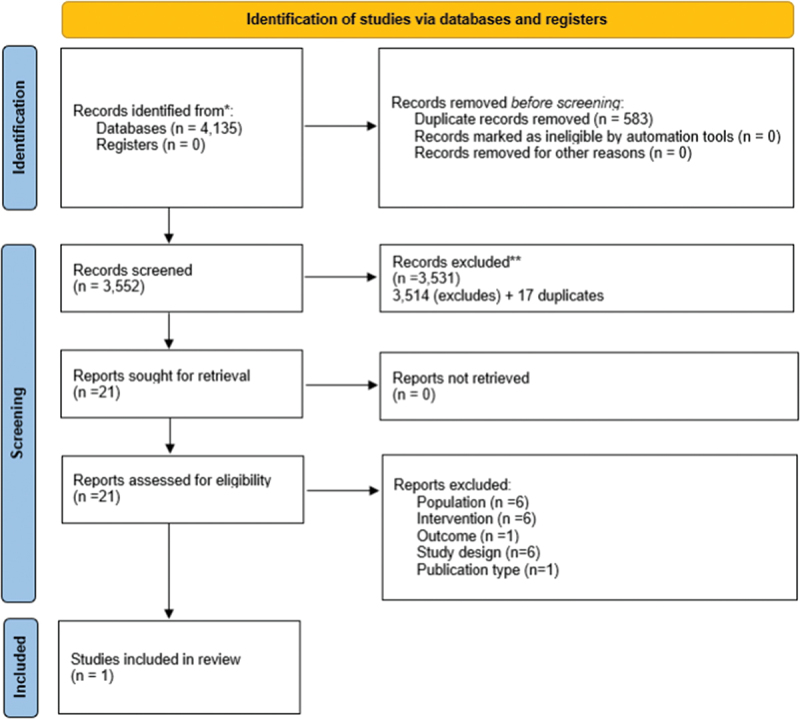
PRISMA diagram of study selection.

This review identified only one published research paper which met the eligibility criteria for inclusion (Johnson et al., 2019). This highlights a paucity of high-quality economic evidence generalisable to different settings and emphasises a need for robust evidence to be generated to ensure decision-makers can implement cost-effective mental health interventions in the prison population.

The single identified study, conducted by Johnson et al. (2019), was a cost-effectiveness study of interpersonal psychotherapy (IPT) among 181 inmates with major depressive disorder (MDD) in the United States. The authors concluded that IPT is both effective and cost-effective and recommend its use for MDD prisoners. However, the limited sample size, short 3-month follow-up period and presence of baseline imbalances (rates of borderline personality disorder and social support) are seen to limit the validity of research findings. Owing to heterogeneity among prison settings and populations, a single research study is insufficient to guide decision-making as there may be limited applicability to different settings and/or populations.

To address the limited evidence base and to ensure economic evaluations can be conducted in future, researchers of mental health interventions should aim to incorporate economic evaluation during the early stages of their research design. This includes considering both intervention costs and wider healthcare resource use which may be affected, while also ensuring that chosen healthcare outcomes are useful to decision-makers. This is particularly important as information on resource use, or costs or cost-effectiveness data cannot often be acquired reliably when collected retrospectively. The inclusion of measures that allow for the generation of quality-adjusted life years (QALYs), which are commonly used in economic evaluation, will enable comparison of outcomes across various disease areas and against pre-determined willingness to pay thresholds (the maximum cost a health system is willing to pay per health outcome) (Whitehead & Ali, 2010). Therefore, it is the responsibility of trial teams to ensure that their methodology is both robust and reliable for producing health economic evidence.

It is hoped that this letter will serve as a reminder to researchers in this field to incorporate an economic component within their work. Clinical evidence alone is often insufficient for decision makers to make informed decisions surrounding funding allocations, something which is of paramount important given the often limited budgets available for healthcare delivery in prisons. The generation of additional economic evidence will not only help to address the identified literature gap but also ensure that funded interventions are cost-effective, thereby improving outcomes for both prisoners and society.
